# Supplementary material for: Comparative multiomics analysis of cell physiological state after culture in a basket bioreactor
Source: Sci Rep. 2022 Nov 23;12:20161. doi: 10.1038/s41598-022-24687-4 (PMC9686226; doi:10.1038/s41598-022-24687-4)
Supplement: Supplementary file 1 — Supplementary Information 1. [file 41598_2022_24687_MOESM1_ESM.zip › raw data/Proteomic raw data/6.KEGGenrichment/EG.vs.CG/ALL/pathway/src/00790.html]

map00790


- K01077, K01113, 3.1.3.1, R04620"

- Down regulated proteins

A0A0D9S8B5(Description:Alkaline phosphatase;Gene:ALPL;log2FC:-1.2816)

Close
